# Transmembrane protein 63A is a partner protein of *Haemonchus contortus* galectin in the regulation of goat peripheral blood mononuclear cells

**DOI:** 10.1186/s13071-015-0816-3

**Published:** 2015-04-09

**Authors:** Cheng Yuan, Hui Zhang, Wang Wang, Yan Li, RuoFeng Yan, LiXin Xu, XiaoKai Song, XiangRui Li

**Affiliations:** College of Veterinary Medicine, Nanjing Agricultural University, Nanjing, 210095 People’s Republic of China

**Keywords:** Galectin, *Haemonchus contortus*, Partner protein, TMEM63A

## Abstract

**Background:**

Hco-gal-m and -f were two isoforms of galectin cloned from male and female *Haemonchus contortus*, respectively, and it was demonstrated that recombinant Hco-gal-m and -f could act as immune suppressors. However, little is known about the receptors or binding partners of these galectins in the host. The research of the molecular mechanisms that govern the interactions between these galectins and host molecules will fill a gap in our understanding how parasite galectins interact with host cells.

**Methods:**

A yeast two-hybrid system was used to identify the binding partners of Hco-gal-m and -f in this research. The interaction between rHco-gal-m and candidate binding protein was validated by co-immunoprecipitation. The localization of transmembrane protein 63A (TMEM63A) in peripheral blood mononuclear cells (PBMCs) was detected by immunofluorescence. The distribution of TMEM63A in T cells, B cells and monocytes in PBMCs was detected by flow cytometry. The immunomodulatory effects of Hco-gal-m and TMEM63A on cell proliferation, migration, apoptosis, nitric oxide production and cytokine secretion were observed by co-incubation of rHco-gal-m and TMEM63A-siRNA with goat PBMCs and monocytes.

**Results:**

We found that TMEM63A, a functionally unknown protein, from goat PBMCs could bind to Hco-gal-m and -f. Immunofluorescence showed that TMEM63A was localized to the cell membrane. Flow cytometric analysis revealed that TMEM63A was expressed in the majority of goat PBMCs. After using RNA interference to knockdown expression of TMEM63A, the PBMC proliferation and migration were significantly increased, while the influence of rHco-gal-m on monocyte phagocytosis, PBMC nitric oxide production and migration were potently blocked. In addition, the production of IL-10, IFN-γ and TGF-β induced by rHco-gal-m were also altered.

**Conclusions:**

Our results show that TMEM63A is a binding partner of Hco-gal-m/f, and involved in the immune responses of host PBMCs induced by Hco-gal-m for the first time.

**Electronic supplementary material:**

The online version of this article (doi:10.1186/s13071-015-0816-3) contains supplementary material, which is available to authorized users.

## Background

Galectins are β-galactoside-binding lectins that are present not only in the cytosol and nucleus but also in the extracellular space [1-3]. Secreted galectins of mammalian can bind to the cell surface and exert a range of functions, including leading to cell adhesion, activating signaling pathways of functional relevance in the control of receptor endocytosis, host-pathogen interactions, and activation and homeostasis of immune cells [4-7]. It has been demonstrated that the interaction of galectin with its receptor on the target cell governs the function of the galectin. The carbohydrate-dependent binding of galectin-4 secreted by intestinal epithelial cells to the CD3 epitope is fully functional and inhibited T cell activation, cycling and expansion [8]. Galectin-8 interacts with several members of the integrin family and modulates the interactions of integrin with the extracellular matrix and thus regulates cell adhesion and cell survival [9]. Thurston et al. suggest that galectin-8 serves as a versatile receptor for vesicle-damaging pathogens in the cytosol [10]. Galectin-9 and galectin-1 both kill thymocytes, peripheral T cells, and T cell lines. However, Bi et al. have found that galectin-9 and galectin-1 require different glycan ligands and glycoprotein receptors to trigger T cell death [11]. Galectin-3 and galectin-9 can both recognize *Leishmania major* (*L. major*) by binding to the *L. major*-specific polygalactosyl epitope, but only galectin-9 can promote the interaction between *L. major* and macrophages [12,13].

Galectin-like proteins have also been identified in parasitic nematodes, cestodes and trematodes, such as *Onchocerca volvulus*, *Teladorsagia circumcincta*, *Haemonchus contortus*, *Trichostrongylus colubriformis*, *Taenia serialis* and *Fasciola hepatica* [4]. Galectins secreted or excreted by parasitic nematodes or other helminths can significantly modulate the host immune response [14-16]. However, little is known about the receptors or binding partners of these galectins in the host. In this case, the research of the molecular mechanisms that govern the interactions between these galectins and host molecules will fill a gap in our understanding how parasite galectins interact with host cells.

*Haemonchus contortus* (*H. contortus*) is one of the most economically important parasites of small ruminants worldwide. Infection can lead to anaemia, loss of condition and death of the host, especially lambs [17,18]. Simultaneously, it can cause immunosuppression and results in a low level immunity in the host [19-21]. This worm is also the most widely used model parasitic nematode in drug discovery [22], vaccine development [23-25] and anthelmintic resistance research [26-29].

Hco-gal-m (Acc. No. AY253330) and Hco-gal-f (Acc.No. AY253331) are two isoforms of galectin which were cloned from male and female *H. contortus* respectively. Our previous studies showed that recombinant Hco-gal-m/f (rHco-gal-m/f) could bind to the surface of goat PBMCs, and function as immune suppressors by inducing cell apoptosis and inhibiting the transcription of IL-1β, IL-4, IFN-γ and TNF-α mRNA in goat PBMCs *in vitro* [30,31]. Our further research indicated that rHco-gal-m/f could influence cell migration, T cell proliferation and differentiation, the expression of MHC-II on monocytes and that of CD25 on T cells [30,32]. Meanwhile, a combined proteomic and transcriptomic analysis revealed that the activations of vascular endothelial growth factor pathway, free radical producing pathway, NFκB pathway and ubiquitin–proteasome pathway in goat PBMC were down-regulated by rHco-gal-m/f [30]. These findings suggested that Hco-gal-m/f were multifunctional molecules that can influence many biological processes, especially those relevant to immune responses or evasion. The discovery of the binding partner of Hco-gal-m/f in goat PBMCs would challenge the current understanding of the *H. contortus* parasite-host interactions.

Transmembrane protein 63A (TMEM63A) is a member of the transmembrane protein family. But its function is still unknown. In the present research, we identified that the effects of Hco-gal-m/f on the proliferation, migration phagocytosis, nitric oxide and some cytokine productions of the goat PBMC were all altered after the TMEM63A (NCBI accession number KF850508) gene was knocked down by specific small interference RNA (siRNA). Our results firstly show that TMEM63A is a binding partner of Hco-gal-m/f, and involved in the immune responses of host PBMCs induced by Hco-gal-m.

## Methods

### Ethics statement

The animals were handled according to the guideline of the Animal Ethics Committee, Nanjing Agricultural University, China. All animal experiments complied with the guidelines of the Animal Welfare Council of China. All experimental protocols were approved by the Science and Technology Agency of Jiangsu Province. The approval ID is SYXK (SU) 2010–0005. The least hardship was certified.

### Animal and cell

Local crossbred goats (3–6-month-old) were fed with hay and whole shelled corn and watered with libitum and housed indoor in pens healthily at Nanjing Agricultural University. All goats were dewormed twice at 2 week intervals with levamisole (8 mg/kg bodyweight) orally at the time of housing to remove naturally acquired strongylid infection [32]. After 2 weeks, a fecal sample from each goat was examined by microscopy for helminth eggs, according to standard parasitological techniques. Goats exhibiting no eggs were used in the subsequent study and daily health observations were performed throughout the experiment.

Goat peripheral venous blood samples were collected from healthy goats consistently. The goat PBMCs were separated from blood of six healthy adult goats with the standard Ficoll-hypaque (GE Healthcare, USA) gradient centrifugation method [33] and were adjusted to a density of 1 × 10^6^ cells/mL in RPMI 1640 or DMEM (GIBCO,UK) containing 10% heat inactivated fetal calf serum (GIBCO, UK), 100 IU/mL penicillin and 100 mg/mL streptomycin (GIBCO, UK) at 37°C in a humidified atmosphere with 5% CO_2_.

Monocytes were isolated by their adherence to plastic surface [34]. The goat PBMCs were seeded in a 6 wells flat-bottom tissue culture plates (Corning, USA) in cell culture medium RPMI 1640 (GIBCO,UK) containing 10% heat inactivated fetal calfserum (GIBCO, UK), 100 U/mL penicillin and 100 mg/mL streptomycin (GIBCO, UK). Plates were incubated at 37°C in a humidified atmosphere with 5% CO_2_ for 1 h [35]. Non-adherent cells were removed by washing twice with phosphate buffered saline (PBS). The adherent cells were collected and adjusted to a density of 1 × 10^6^ cells/mL in cell medium at 37°C in a humidified atmosphere with 5% CO_2_.

Cells used for the experiments were freshly isolated from goat peripheral blood. Cell viability, as determined by trypan blue dye exclusion, was more than 95% in all cases.

### Identification of binding partners for Hco-gal-m and -f by yeast two-hybrid (YTH) screening

Construction of the goat PBMC cDNA library for YTH screening is described in Additional file 1. A split-ubiquitin YTH DUALhunter system (Dualsystems Biotech, Switzerland) was used to identify interaction partners of Hco-Gal-m and -f from goat PBMC. The coding regions of Hco-Gal-m and -f were amplified by PCR using the primers Hco-Gal-F and Hco-Gal-R (Additional file 2: Table S1) from the recombinant plasmid pBV220-*Hco-*gal-m/f that was previously constructed in our laboratory [36]. The PCR product was next inserted into the split-ubiquitin YTH Cub domain vector pDHB1 to generate the bait plasmid, pDHB1-Gal-m/f, which was verified by DNA sequencing. Expression of the bait protein was confirmed. The plasmid pDHB1-Gal-m/f was used to screen a goat PBMC cDNA library to identify Hco-Gal-m- and -f-interacting proteins. Positive yeast clones encoding Hco-Gal-m- and -f-interacting proteins were purified and retested for growth phenotypes. Plasmid DNA preparations for these yeast clones were generated using the Yeast Plasmid Extraction Kit (OmegaBio-tek, Georgia, USA). The insert fragments in these prey plasmids were detected by PCR amplification using the primers pPR3N-F and pPR3N-R (Additional file 2: Table S1). The selected prey plasmids were amplified in DH_5α_, recovered by ampicillin selection and identified by DNA sequencing with the pPR3N-F and pPR3N-R primers from Invitrogen (Life Technologies, Shanghai, China). The DNA sequences were used to search GenBank. After removing duplications, the remaining plasmids were retransformed into yeast cells that contained pDHB1-Gal-m/f to retest the interactions with Hco-Gal-m and -f in yeast. LargeT was used as a bait control and Alg5 fused to NubG or NubI was used as the negative or positive prey control, respectively.

### Validation of the interaction between rHco-gal-m and TMEM63A by co-immunoprecipitation (co-IP) and immunoblotting

To validate the interaction between rHco-gal-m and candidate binding protein, 20 μL rHco-gal-m at a concentration of 1 μg/μL was added to each well containing goat PBMC (1 × 10^6^ cells per well), and then incubated for 12 h. In each IP experiment, 5× 10^7^ PBMCs were pelleted and lysed in 4 mL lysate buffer (50 mM Tris, 150 mM NaCl, 1% Triton X-100, 1% NP-40, 1 mM EDTA, 50 mM NaF, and 40 mM sodium pyrophosphate, pH 7.4) [37], containing a protease inhibitor cocktail (Merck, Darmstadt, Germany). Cell lysates were precleared by adding 1 μg rat normal IgG and 20 μL Protein A/G PLUS-Agarose beads (Santa Cruz Biotechnology, Texas, USA), and then incubated at 4°C for 30 min. After pelleting beads by centrifugation at 1,000 × g for 5 min at 4°C, the protein concentration in the supernatant (or the cell lysate for IP) was determined using the Pierce™ BCA™ Protein Assay (Thermo Fisher Scientific, MA, USA).

In the forward IP experiment, triplicate 1 mg lysates were each separately incubated overnight at 4°C with the following: rat anti-TMEM63A-NO IgG for input samples; rat anti-Hco-Gal IgG for IP of TMEM63A; or normal rat IgG for negative control samples. Immune complexes were isolated using 20 μL protein A/G plus agarose following the manufacturer’s protocol and were then analyzed with rat anti-TMEM63A-NO IgG by immunoblotting.

In the reverse IP experiment, triplicate 1 mg lysates were each incubated separately overnight at 4°C with the following: rat anti-Hco-Gal IgG for input samples; rat anti-TMEM63A-NO IgG for IP of rHco-gal-m; or normal rat IgG for negative control samples. Immune complexes were isolated using 20 μL protein A/G plus agarose and were then analyzed with rat anti-Hco-Gal IgG by immunoblotting. Antibodies and the production of antibodies used in these experiments are described in the Additional file 1, Additional file 2: Table S2 and Additional file 3, Additional file 4, Additional file 5, Additional file 6.

Input, IP and negative control samples in SDS loading buffer were loaded onto a SDS–PAGE gel (15% acrylamide gels, 15 μL/well) and run at 110 V. Proteins on the gel were transferred onto a 0.2 μm PVDF transfer membrane (Thermo Fisher Scientific, MA, USA) at 400 mA for 1 h. Then, the blotting membrane was blocked with 5% skim milk/TBST (Tris-buffered saline containing 0.1% Tween-20) for 1 h at room temperature before probing with rat anti-TMEM63A-NO IgG or rat anti-*Hco*-Gal IgG (all dilutions, 1:1000) overnight at 4°C. Following a wash step with TBST, bound antigen–antibody complexes were detected using chicken anti-rat IgG conjugated to horseradish peroxidase (HRP) antibody (dilutions, 1:5000; Santa Cruz Biotechnology, Texas, USA). Reactions were detected using an enhanced HRP-DAB Chromogenic Substrate Kit (TIANGEN BIOTECH, Beijing, China) according to the manufacturer’s instructions.

### Detection of the localization of TMEM63A in PBMCs by immunofluorescence (IF)

Freshly isolated PBMCs from goat blood were used for the IF assay. The IF analyses were performed on 4% paraformaldehyde-fixed PBMCs (10^5^ cells/sample) plated on 0.01% poly-L-lysine-coated cover slips. Then, cells were permeabilized by incubation for 5 min in 0.5% Triton X-100 in PBS, and were treated with a blocking solution (2% BSA in PBS) for 30 min to reduce background staining. After sequential incubation with the rat anti-TMEM63A-NO IgG (0.5 μg) or negative rat IgG (0.5 μg, for negative control), respectively, for 2 h and incubation with secondary antibody coupled to the Cy3 (Beyotime, Jiangsu, China) fluorescent dye (1:300) for 1 h, 3,3’-Dioctadecyloxacarbocyanine (DiOC18(3), 5 μM; Beyotime, Jiangsu, China) and 2-(4-Amidinophenyl)-6-indolecarbamidine dihydrochloride (DAPI, 1.5 μM; Sigma, MO, USA) were used for plasma membrane and nucleus staining, respectively, for 6 min each. Then, protein localization was determined by observing the staining patterns with a 100× oil objective lens on a laser scanning confocal microscope (LSM710, Zeiss, Jena, Germany). Exposure conditions were applied uniformly for each color channel. All procedures were carried out at room temperature. Digital images were captured using the Zeiss microscope software package ZEN 2012 (Zeiss, Jena, Germany).

### Detection of the distribution of TMEM63A in T cells, B cells and monocytes in PBMCs by flow cytometry

Freshly isolated PBMCs from goat peripheral blood were used for this experiment. Cell surface staining (10^6^ cells/reaction) was carried out with the following antibodies that cross-react with the respective goat antigens, according to the manufacturer’s instructions: mouse anti-bovine CD2-FITC (1 μg, AbDserotec, BioRad Laboratories, CA, USA) to detect T cells, mouse anti-bovine CD21-FITC (1 μg, AbDserotec, BioRad Laboratories, CA, USA) to detect B cells, and mouse anti-bovine CD14-FITC (1 μg, AbDSerotec, BioRad Laboratories, CA, USA) to detect monocytes. After incubation with antibodies at 4°C for 30 min, cells were treated with FIX & PERM® cell permeabilization reagents (Multi sciences, Zhejiang, China) and washed with 3 mL PBS (pH 7.4) containing 2% FBS. To label the target cells, rat anti-TMEM63A-NO IgG (1 μg) were incubated with cells at room temperature for 1 h, followed by incubation with chicken anti-rat IgG-PE (1:300, at room temperature for 30 min, SantaCruz Biotechnology, Texas, USA). Normal mouse IgG_1_-FITC (1 μg, SantaCruz Biotechnology, Texas, USA) and negative rat IgG (1 μg) were used to set a ‘fluorescence minus one’ control. As controls for partial analog compensation, individual goat PBMC samples were stained separately using each fluorochrome individually. Samples were analyzed on a FACS Calibur™ flow cytometer (BD Biosciences, CA, USA). Overall, at least 10,000 events in the PBMC gate were collected for each sample. The gating was standardized and set using ‘fluorescence minus one’ controls. Data were analyzed using FlowJo 7.6 software (Tree Star, OR, USA).

### Small interferon RNA

Three small interfering RNA (siRNA) were designed to knockdown *TMEM63A* gene (Additional file 2: Table S3). TMEM63A-siRNA-1 showed the highest interference efficiency and were selected for use in further experiments (Additional file 1 and Additional file 7: Figure S5). The siRNAs used in this study were chemically synthesized by Invitrogen (Life Technologies, Shanghai, China) and dissolved in RNase-free water to 20 μM. The suitable time for interference was also determined and is detailed in the Additional file 1 and Additional file 7: Figure S5. The non-specific siRNA (ns siRNA) sequences used in this study are listed in Additional file 2: Table S3.

### Cell treatment

After the goat PBMCs or monocytes were isolated from peripheral venous blood, cells were treated with two different kinds of incubation periods. The cell incubation periods were exhibited by siRNA transfection and rHco-gal-m stimulation to show the possible contribution to goat PBMCs made by TMEM63A and rHco-gal-m. The efficiency RNA interference (RNAi) transfection system used here was optimized. The concentrations of rHco-gal-m (40 μg/mL) used here was based on previous dose response studies that produced remarkable biological response without causing toxicity to the cells [31,38,39]. Cells for RNA interference period can be shown as blank group (group 1), ns siRNA group (group 2), ns siRNA/g group (group 3), 63A-siRNA group (group 4) and 63A-siRNA/g group (group 5),which were incubated with equal volume reduced serum medium (group 1), ns siRNA (group 2 and 3) and TMEM63A-siRNA-1 (group 4 and 5), for 60 h with the cell concentration of 1 × 10^6^/mL. RHco-gal-m in all RNA interference groups (group 3 and 5) was added 12 h before the end of RNA interference period.

### Cell proliferation assay

According to the manufacturer’s instructions, 100 uL cell counting kit-8 assay reagent (Beyotime Biotechnology, China) was added in each well of 96-well plates and incubated for 4 h at 37°C in a humidified atmosphere with 5% CO_2_ away from light at the end of the RNA interference incubation period. The absorbance of the colored solution was measured using a microplate reader (Bio-Rad Laboratories, USA) at a test wavelength of 450 nm (OD450). Cells in blank group were served as controls and the OD450 were set as 100%. Cell proliferation index was calculated by the formula: OD450 group/OD450 control.

### Cell phagocytosis assay

The monocytes detected here were treated with siRNA and rHco-gal-m in advance as described previously. Before being added in a 96-well plate (200 μL/well), the cells were washed with fresh medium and were adjusted to a density of 1 × 10^5^ cells/mL. Subsequently, the monocytes were incubated with 50 μL 0.1% neutral red for 2 h at 37°C in a humidified atmosphere with 5% CO_2_, then were washed twice with PBS. Eventually, neutral red was extracted with 100 μL NaH_2_PO4 (0.05 mol/L) in 50% ethanol [40]. The absorbance of the colored solution was measured using a microplate reader (Bio-Rad Laboratories, USA) at a test wavelength of 540 nm (OD540). Cells in blank group were served as controls and the OD540 were set as 100%. Cell phagocytosis index was calculated by the formula: OD540group/OD540 control.

### Measurement of nitric oxide production

The goat PBMCs were harvested and washed twice with PBS at the end of the RNA interference period. Then the cells from different groups were plated in 96-well plates in DMEM medium in a density of 1× 10^6^ cells/mL. Production of nitric oxide by PBMCs was determined by measurement of intracellular nitrite in the PBMC by using the Griess assay [41] according to the instruction of Total Nitric Oxide Assay Kit (Beyotime Biotechnology, China). Absorbance of the colored solution at 540 nm (OD540) in each well was measured using a plate reader (Bio-Rad Laboratories, USA). Absorbance values were converted to micromoles per liter using a standard curve that was generated by addition of 0 to 80 μmol/L sodium nitrite to fresh culture media.

### Cell migration assay

After achieving gene knockdown, cells seeded in 12-well plates were collected and the density was adjusted to 1.5 × 10^6^ cells/mL. Migration was assayed in Millicell® insert with 8.0 μm pores (Merck-Millipore, USA) [30,35]. Then 200 μL cells with the density of 1.5 × 10^6^ cells/mL were seeded in the upper chamber while the lower chamber was filled with 1300 μL 1640 cell medium. After 2 h of incubation the filters were removed and the cells that migrated through the membrane into the lower chamber were counted with a Neubauer counting chamber. The results were presented as percentages of the seeded PBMC. Each experiment was performed in triplicate.

### Detection of cytokine transcription

After achieving gene knockdown, cells were treated in different ways for 12 h to detect cytokine transcription. In each experiment, 40 μL vehicle (PBS, unstimulated negative control) or 40 μL rHco-gal-m at 1 μg/μL were added to the cells to yield a final volume of 1 mL per well.

Treated PBMCs were collected for total RNA extraction. Genomic DNA contamination in the RNA preparation was removed by treatment with RNase-free DNase I (TaKaRa, Clontech Laboratories, CA, USA). Next, RNA was reverse-transcribed using a PrimeScript™ RT reagent kit (TaKaRa, Clontech Laboratories, CA, USA) according to the manufacturer’s instructions. The cDNA were used to measure cytokine expression by real-time PCR.

PCR reactions and conditions are described in Additional file 1. Primers for real-time PCR are listed in Additional file 2. The stability of beta-actin expression, used as an endogenous reference gene, was verified. The amplification efficiencies of all target and endogenous reference genes were examined using real-time PCR, and these amplification efficiencies were approximately similar (Additional file 2: Table S4). We considered real-time PCR assays that generated efficiencies in the range of 96-104% to be acceptable for use in our assays. Each sample was run in technical triplicates and a non-template control was included. A melting curve was generated after completion of the thermal PCR program to check for the presence of one gene-specific peak and the absence of primer dimer. Raw cycle thresholds (C_t_), obtained from ABI Prism 7500 software version 2.0.6 (Applied Biosystems, CA, USA), were used in the comparative C_t_ method (the 2^-ΔΔCt^ method) [42].

### Statistical analysis

Data are expressed as the mean ± the standard deviation (SD) of the mean. One-way analysis of variance was performed using the GraphPad Premier 5.0 software package (GraphPad Prism, CA, USA) to compare averages between groups. Significance was set at P < 0.001.

## Results

### TMEM63A is a binding protein for Hco-Gal-m and -f

The YTH system was used to identify binding partners of Hco-Gal-m and -f from a goat PBMC cDNA library. The resulting library contained at least 2 × 10^6^ primary recombinants, and the average insert size was 1.0 kb. In the YTH screen, 81 clones encoding proteins that showed a potential interaction with the Hco-Gal-m and -f proteins in yeast cells were identified. Multiple potential Hco-Gal-m- and -f-interacting proteins were identified in the retest. Then, these gene products were identified by DNA sequencing and searches of GenBank. One of the gene products was determined to be TMEM63A (NCBI accession number KF850508). TMEM63A is a novel protein that has not been characterized by any functional studies to date, so TMEM63A was chosen to proceed further research. Meanwhile, because the same candidate proteins were identified using either Hco-Gal-m or Hco-Gal-f as bait, only rHco-Gal-m was used in subsequent experiments as a representative protein.

### Co-IP assays demonstrated that rHco-Gal-m could bind to TMEM63A

**Figure 1:**
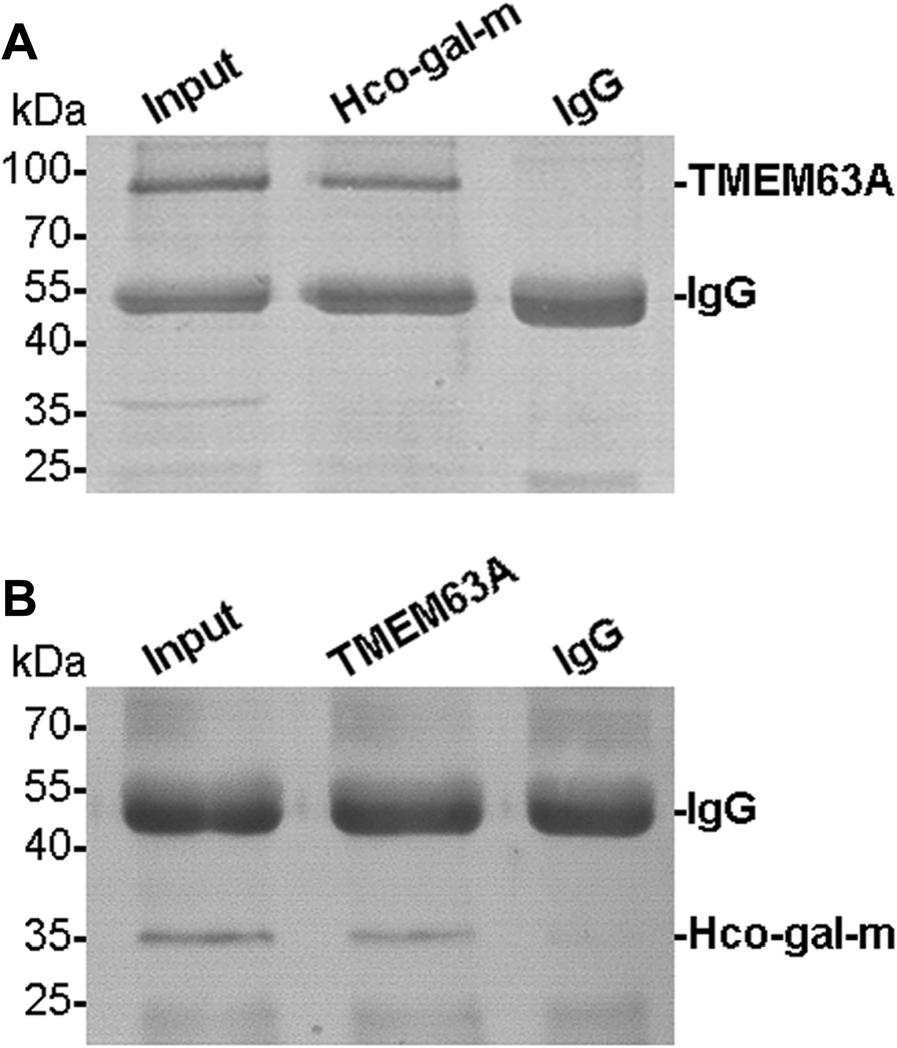
**Co-IP assays indicate that rHco-Gal-m can bind to TMEM63A. (A)**: Lane, Input: Cell lysates were precipitated with rat anti-TMEM63A-NO IgG. Lane, Hco-Gal-m: Cell lysates were precipitated with rat anti-Hco-Gal IgG. Lane, IgG: Cell lysates were precipitated with rat normal rat IgG. Immunoblot analysis using rat anti-TMEM63A-NO IgG (TMEM63A) demonstrated that rHco-Gal-m could bind to TMEM63A. **(B)**: Lane, Input: Cell lysates were precipitated with rat anti-Hco-Gal IgG. Lane, TMEM63A: Cell lysates were precipitated with rat anti-TMEM63A-NO IgG. Lane, IgG: Cell lysates were precipitated with normal rat IgG. Immunoblot analysis using rat anti-Hco-Gal IgG demonstrated that rHco-Gal-m could bind to TMEM63A.

The results obtained using the YTH system were confirmed in rHco-Gal-m-stimulated (12 h) goat PBMC by two independent co-IP approaches. TMEM63A were detected in rHco-Gal-m immune complexes (IP) and in the PBMC lysates (Input), but not in the rat normal IgG control (IgG) group (Figure 1A). Reciprocally, a reverse co-IP assay using specific antibodies rat anti-TMEM63A-NO IgG (Figure 1B) followed by western blotting confirmed the binding of TMEM63A to rHco-Gal-m. The results of the co-IP assays strongly suggested that the interactions of Hco-Gal-m with TMEM63A in PBMCs indicated specific binding.

### TMEM63A was localized to the cell surface in PBMCs

**Figure 2:**
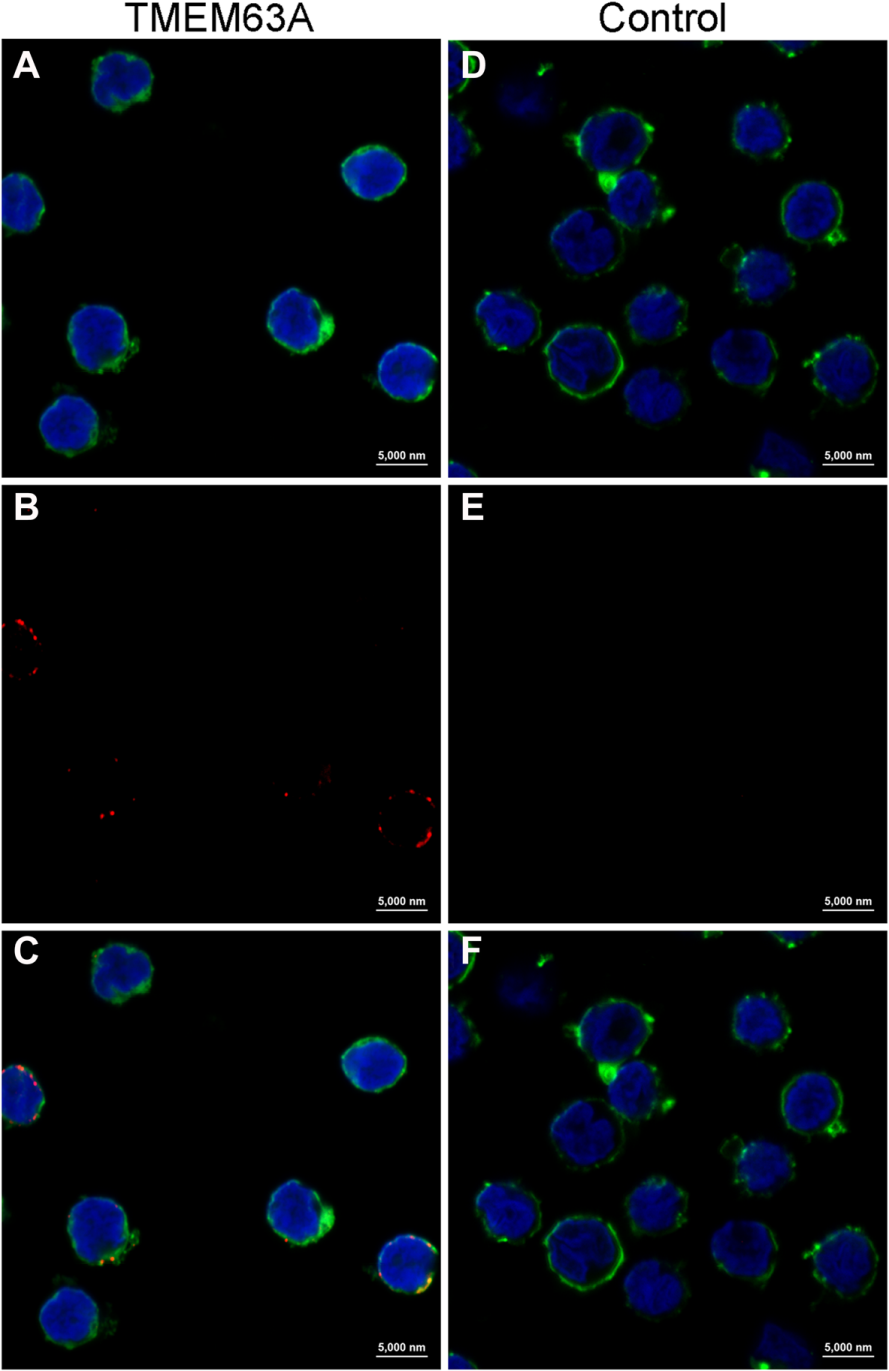
**TMEM63A localized to the cell surface in PBMCs.** Goat PBMC were first fixed and then permeabilized with detergent prior to IF analysis. Then, cells were incubated with rat anti-TMEM63A-NO IgG (TMEM63A) or negative rat IgG (Control). DIO (green), DAPI (blue) and Cy3-conjugated secondary antibodies (red) were used for triple staining. **(A and D)**: cell membrane (green) and nuclei (blue) staining of cells; **(B and E)**: staining of target proteins (red); **(C and F)**: a merged image of the three colors. TMEM63A only localized to the cell membranes. Scale bars represent 5000 nm.

We observed the locations of TMEM63A in intact and permeabilized PBMCs by IF. Cellular membranes were stained with VybrantDiO and then confocal imaging was used to visualize both the membrane and sphere locations [43]. Nuclei were stained with DAPI to observe the nuclear morphology [44]. Confocal microscopy images showed that TMEM63A only localized to the cell surface (Figure 2B and C). In the control group, no red fluorescence was observed (Figure 2E and F).

### TMEM63A is expressed in T cells, B cells and monocytes of PBMCs

**Figure 3:**
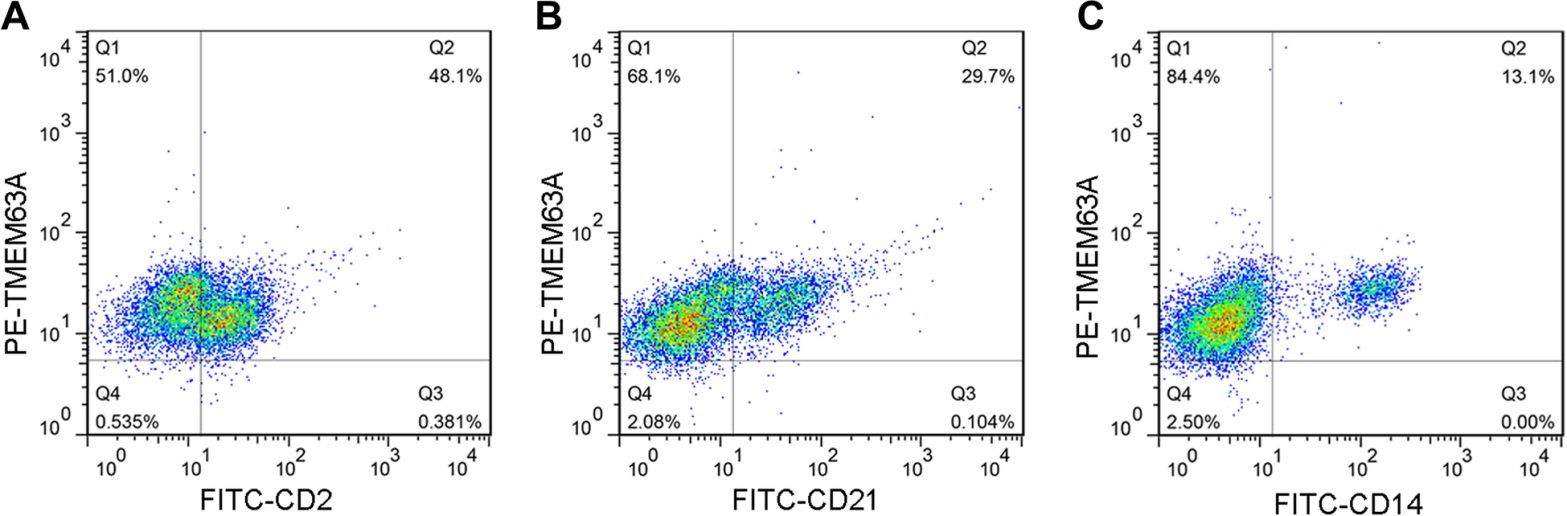
**Analysis of TMEM63A expression in goat PBMCs by flow cytometry.** T cell **(A)**, B cell **(B)** and monocyte **(C)** populations were identified using FITC-CD2, FITC-CD21 and FITC-CD14 (x-axis). Cells that expressed TMEM63A were identified using rat anti-TMEM63A-NO IgG, followed by incubation with chicken anti-rat IgG-PE (y-axis). **(A)**: Q1: TMEM63A^+^/CD2^−^ cells; Q2: TMEM63A^+^/CD2^+^ cells; Q3: TMEM63A^−^/CD2^+^ cells; Q4: TMEM63A^−^/CD2^−^ cells. **(B)**: Q1: TMEM63A^+^/CD21^−^ cells; Q2: TMEM63A^+^/CD21^+^ cells; Q3: TMEM63A^−^/CD21^+^ cells; Q4: TMEM63A^−^/CD21^−^ cells. **(C)**: Q1: TMEM63A^+^/CD14^−^ cells; Q2: TMEM63A^+^/CD14^+^ cells; Q3: TMEM63A^−^/CD14^+^ cells; Q4: TMEM63A^−^/CD14^−^ cells. The percentages of cells with different staining patterns are shown. The results presented here are from an independent experiment that is representative of three independent experiments.

The frequencies of TMEM63A^+^ T cells (TMEM63A^+^/CD2^+^, 48.100%) were approximately similar to the frequency of total T cells (CD2^+^, 48.100% + 0.381%) in PBMCs (Figure 3A). The frequencies of TMEM63A^+^ B cells (TMEM63A^+^/CD21^+^, 29.700%) were approximately similar to the frequency of total B cells (CD21^+^, 29.700% + 0.104%, Figure 3B). TMEM63A^+^ monocytes (TMEM63A^+^/CD14^+^, 13.100%) were approximately 100% of total monocytes (CD14^+^, 13.100% + 0.000%) in PBMCs (Figure 3C). These results indicate that the majority of goat PBMCs expressed TMEM63A

### Knockdown of the *TMEM63A* gene and rHco-gal-m-treatment affected the PBMC proliferation

**Figure 4:**
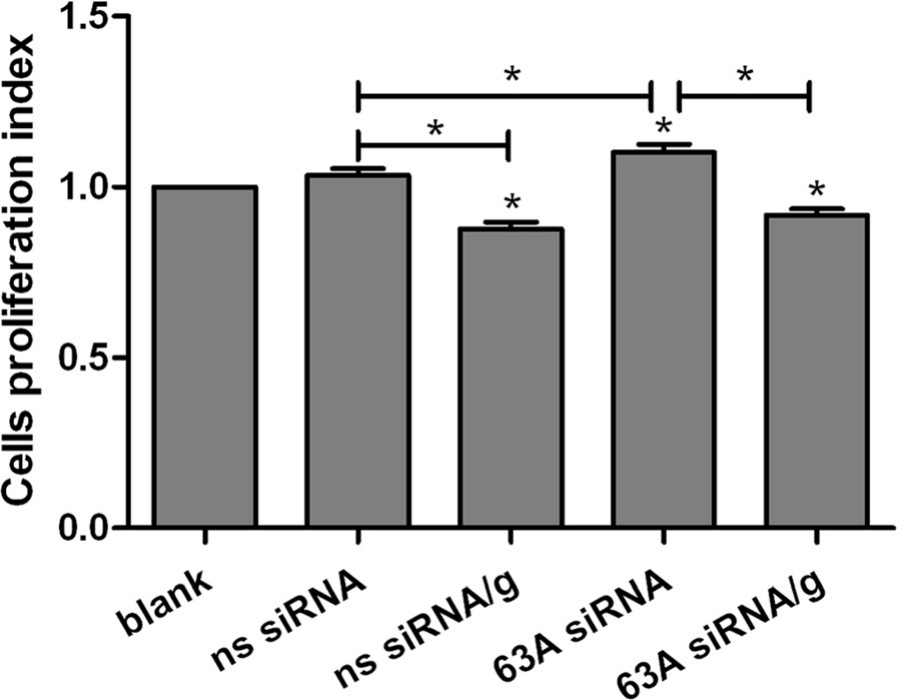
**Knockdown of the TMEM63A gene and rHco-gal-m-treatment affected the PBMC proliferation.** PBMC can be share as blank group (group 1), ns siRNA group (group 2), ns siRNA/g group (group 3), 63A siRNA group (group 4) and 63A-siRNA/g group (group 5), which were incubated with equal volume reduced serum medium (group 1), ns siRNA (group 2 and 3) and TMEM63A-siRNA-1 (group 4 and 5) for 60 h. RHco-gal-m in all RNA interference groups (group 3 and 5) was added 12 h before the end of RNA interference period. The proliferation was measured by CCK-8 incorporation. Cells proliferation index was calculated considering the OD450 values in blank group as 100%. Results presented here were collected from one independent experiment and were representative of three independent experiments. Data were represented as mean ± SD, n = 6; ^*^p < 0.001 versus the ns siRNA group; an asterisk and a capped line designates two groups differ significantly (p < 0.001).

As demonstrated by incorporation of cell counting kit-8 (CCK-8), no PBMC multiplication was induced by the ns siRNA in ns siRNA group compared with blank group (Figure 4). rHco-gal-m-treatment significantly suppressed the proliferation of PBMC in the ns siRNA/g group and 63A siRNA/g group compared with ns siRNA group and 63A siRNA group (Figure 4). No significant difference was observed between ns siRNA/g group and 63A siRNA/g group (Figure 4). After the TMEM63A siRNA-treatment, the PBMC proliferation significantly increased in the 63A siRNA group compared with the ns siRNA group (Figure 4).

### Knockdown of the TMEM63A gene and rHco-gal-m-treatment affected the monocyte phagocytosis

**Figure 5:**
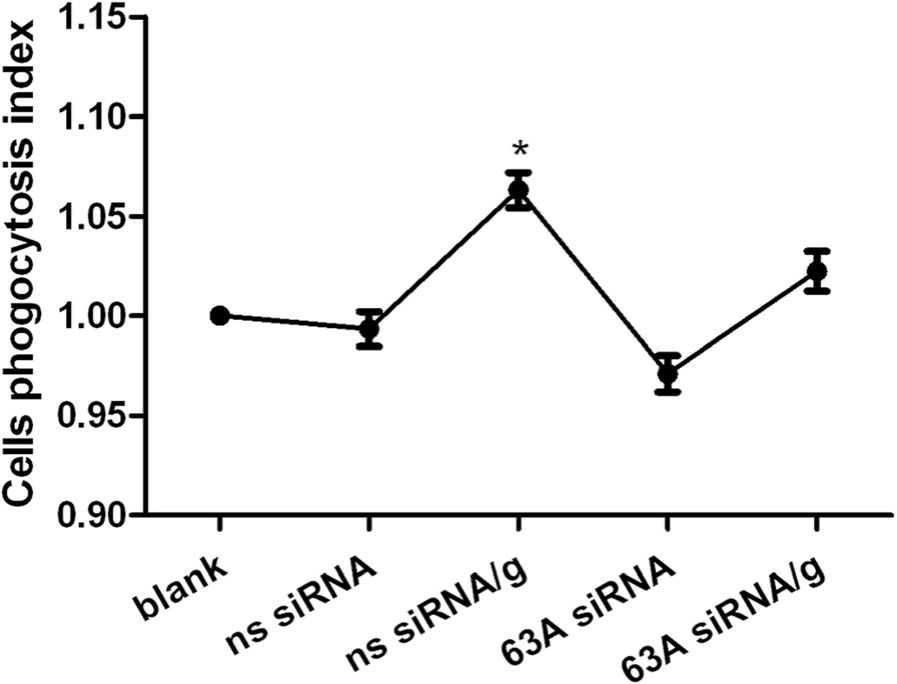
**Knockdown of the TMEM63A gene and rHco-gal-m-treatment affected the monocytes phagocytosis.** Monocytes can be share as blank group (group 1), ns siRNA group (group 2), ns siRNA/g group (group 3), 63A siRNA group (group 4) and 63A siRNA/g group (group 5), which were incubated with equal volume reduced serum medium (group 1), ns siRNA (group 2 and 3) and TMEM63A-siRNA-1 (group 4 and 5) for 60 h. RHco-gal-m in all RNA interference groups (group 3 and 5) was added 12 h before the end of RNA interference period. Cells phagocytosis index was calculated considering the OD540 values in blank group as 100%. Results presented here were collected from one independent experiment and were representative of three independent experiments. Data were represented as mean ± SD, n = 4; ^*^p <0.001 versus the ns siRNA group.

To confirm the impact of TMEM63A knockdown and rHco-gal-m-treatment on monocyte phagocytosis, a cell phagocytosis assay was performed. When monocyte were incubated with rHco-gal-m, the phagocytosis of monocyte in ns siRNA/g group was significantly increased compared to the ns siRNA group (Figure 5). No significant difference was observed between ns siRNA group and 63A siRNA group or 63A siRNA/g group (Figure 5).

### Knockdown of the TMEM63A gene and rHco-gal-m-treatment affected the PBMC nitric oxide production

**Figure 6:**
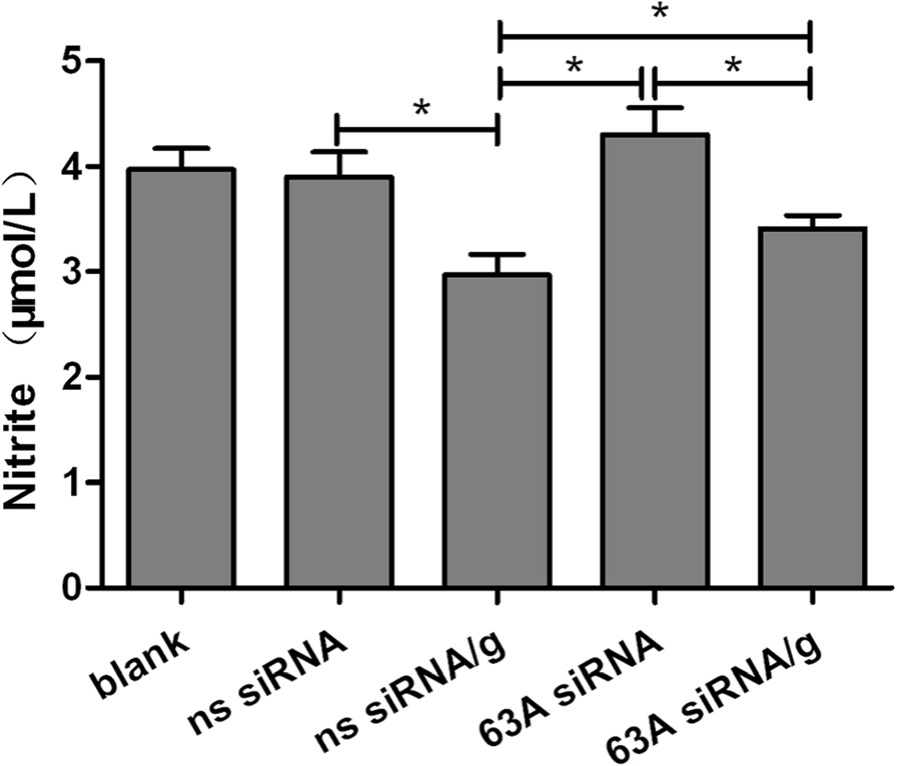
**Knockdown of the TMEM63A gene and rHco-gal-m-treatment affected the PBMC nitrite oxide production.** PBMC can be share as blank group (group 1), ns siRNA group (group 2), ns siRNA/g group (group 3), 63A siRNA group (group 4) and 63A siRNA/g group (group 5), which were incubated with equal volume reduced serum medium (group 1), ns siRNA (group 2 and 3) and TMEM63A-siRNA-1 (group 4 and 5) for 60 h. RHco-gal-m in all RNA interference groups (group 3 and 5) was added 12 h before the end of RNA interference period. The nitrite concentration in the PBMCs was measured by using the Griessassay and used as an indicator of nitric oxide production by the PBMCs. Results presented here were collected from one independent experiment and were representative of three independent experiments. Data were represented as mean ± SD, n = 4; an asterisk and a capped line designates two groups differ significantly (p < 0.001).

We measured nitric oxide production by TMEM63A gene knockdown PBMC treated with rHco-gal-m. Treatment with rHco-gal-m significantly suppressed nitric oxide production in the ns siRNA/g group and 63A siRNA/g group compared with ns siRNA group and 63A siRNA group (Figure 6). In the rHco-gal-m-treatment cells, the nitric oxide production in the 63A siRNA/g group was significantly increased compared to the cells in the ns siRNA/g group (Figure 6). After the TMEM63A siRNA-treatments, there was no influence on the PBMC nitric oxide production in the 63A siRNA group compared with the ns siRNA group (Figure 6).

### Knockdown of the TMEM63A gene and rHco-gal-m-treatment affected PBMC migration

**Figure 7:**
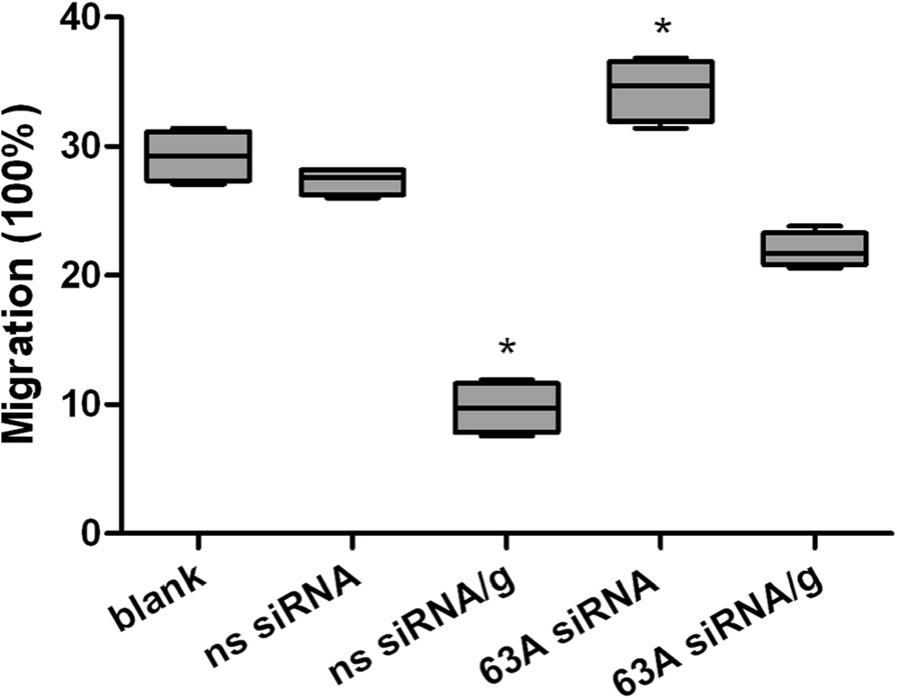
**Knockdown of the TMEM63A gene and rHco-gal-m-treatment affected the PBMC migration.** PBMC can be share as blank group (group 1), ns siRNA group (group 2), ns siRNA/g group (group 3), 63A siRNA group (group 4) and 63A siRNA/g group (group 5), which were incubated with equal volume reduced serum medium (group 1), ns siRNA (group 2 and 3) and TMEM63A-siRNA-1 (group 4 and 5) for 60 h. RHco-gal-m in all RNA interference groups (group 3 and 5) was added 12 h before the end of RNA interference period. Then the random migration was determined. The data are presented asbox-and-whiskers blot, with the box containing 50% of the values, and the whiskers showing the highest and the lowest values. The median is indicated by the horizontal bar, the mean value as square. Results presented here were collected from one independent experiment (n = 4) and were representative of three independent experiments; ^*^p < 0.001 versus the ns siRNA group.

To confirm the impact of TMEM63A knockdown and rHco-gal-m/f-treatment on PBMC migration, a cell migration assay was performed. In the blank group, 29.25 ± 1.98% PBMC migrated into the lower chambers. No significant difference was observed between blank group and ns siRNA group (27.36 ± 1.04%). After the treatment of rHco-gal-m, the number dramatically decreased to 9.75 ± 1.98% (Figure 7). When PBMC were treated with TMEM63A siRNA, the percentage of migrated PBMC was up to 34.40 ± 2.40% in 63A siRNA group, then the number significantly decreased to 21.94 ± 1.36% after the treatment of rHco-gal-min 63A siRNA/g group (Figure 7). Meanwhile, no significant difference was observed between ns siRNA group and 63A siRNA/g group (Figure 7).

### Knockdown of the TMEM63A gene affected the transcription of cytokines in goat PBMCs

**Figure 8:**
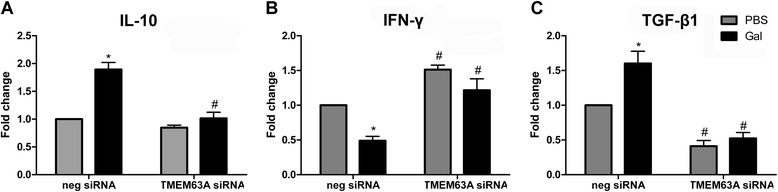
**Relative levels of cytokine mRNA transcripts in goat PBMCs pretreated with negative or** ***TMEM63A*** **siRNA.** Goat PBMCs pretreated with TMEM63A siRNA were stimulated with PBS or r*Hco*-gal-m. After the stimulation, changes in cytokine transcript levels in cells were detected by real-time PCR. Each stimuli is shown in one of the two graphs. **(A)** IL-10; **(B)** IFN-γ; **(C)** TGF-β1. Data are presented as the means ± SD; n = 3. Significant differences between the two stimulation conditions for each RNAi group are indicated by an asterisk (*, p < 0.001). A number sign (#) designates that the mean of the *TMEM63A* knockdown group differs significantly from the mean of the negative siRNA group (p < 0.001) for a stimulation condition. Representative data from one independent experiment with technical triplicates are shown that is representative of three independent experiments.

Goat PBMCs pretreated with ns or TMEM63A siRNA were stimulated by PBS (vehicle) or rHco-gal-m. Then, expression of cytokine mRNA transcripts in PBMCs were detected by real-time PCR. From the result of real-time PCR, IL-10, IFN-γ and TGF-β1 were regulated at a transcriptional level (Figure 8).

In all ns siRNA groups, expression of the IL-10 and TGF-β1 mRNA transcripts were significantly increased by rHco-gal-m compared to the PBS-treated controls (Figure 8A and C). Conversely, the mRNA transcript level of IFN-γ was significantly decreased by rHco-gal-m treatment (Figure 8B).

In the *TMEM63A* knockdown cells, the level of IL-10 mRNA transcripts in the PBS-treated group did not change compared to the level in ns siRNA-treated cells (Figure 8A). Notably, the level of IFN-γ mRNA transcripts in the PBS-treated group was significantly increased compared to the levels in the ns siRNA-treated cells (Figure 8B). Whereas the level of TGF-β1 mRNA transcripts in the PBS-treated group was significantly reduced compared to the levels in the ns siRNA-treated cells (Figure 8C). In the *TMEM63A* knockdown cells, the levels of IL-10 and TGF-β1 mRNA transcripts in the rHco-gal-m-treated group were significantly decreased compared to the ns siRNA-treated cells, and the level of IFN-γ mRNA transcripts in the rHco-gal-m-treated group was significantly increased (Figure 8).

## Discussion

In this study, using YTH screening and co-IP assays, we found for the first time that TMEM63A was a binding protein for Hco-gal-m and -f. IF microscopy showed that TMEM63A was located on the plasma membrane of the cells. After knockdown of the genes that encode *TMEM63A* by RNAi, the influence of rHco-gal-m on the proliferation, phagocytosis, nitric oxide production, migration and cytokines expression of the siRNA-treated cells were changed.

TMEM63A is a novel protein that has not been characterized by any functional studies to date. In the present study, we cloned the nucleotide sequence of goat TMEM63A and analyzed its structures for the first time (unpublished data). The translated amino acid sequence of TMEM63A suggested that it was a polytopic membrane protein and was largely embedded within the membrane. A possible function in signal transduction could be inferred from its multiple transmembrane structures. By staining TMEM63A with anti-TMEM63A-NO IgG, which was directed against the N-terminal domain of this protein, we showed that TMEM63A was present on the cell surface (Figure 2), indicating that TMEM63A might be a receptor for Hco-gal-m on goat PBMC membrane. However, the nature of the TMEM63A should be further investigated.

It was reported that the C-terminal carbohydrate recognition domain (CRD) of galectin-1 and galectin-9, but not N-terminal CRD, was the primary determinant of receptor recognition, death pathway signaling, and target cell susceptibility [11]. It was also noted that the two CRDs of Hco-Gal-m and -f show different carbohydrate binding and hemagglutination abilities [45]. Based on the prediction of N-linked glycosylation sites using the NetNGlyc 1.0 server (http://www.cbs.dtu.dk/services/NetNGlyc/), potential N-linked glycosylation sites are indeed present in the protein sequence of TMEM63A. Therefore, interactions between Hco-Gal-m and -f and TMEM63A might occur at glycosylation sites that are recognized by Hco-Gal-m and -f. The domains or structures of Hco-Gal-m and -f that dominate the binding of Hco-Gal-m and -f to TMEM63A, and the relationship between the CRDs and such domains or structures require further studies.

Recently, it was demonstrated that the carbohydrate-dependent interaction of galectin-1 and −3 with CD6 and CD166/ALCAM could modulate T cell proliferation and cell adhesion phenomena mediated by the CD6-CD166/ALCAM pair [46]. In this research, rHco-gal-m-treatment significantly suppressed the proliferation of PBMC in the ns siRNA-treated cells (Figure 4), which was consistent with the results of previous studies [32]. Meanwhile, after the TMEM63A siRNA-treatment, the PBMC proliferation significantly increased in the 63A siRNA group compared with the ns siRNA group (Figure 4). However, no significant difference was observed between ns siRNA/g group and 63A siRNA/g group (Figure 4). These findings indicated that TMEM63A might participate in the modulation of cell proliferation in PBMC, but not in the influence of cell proliferation induced by rHco-gal-m. So there should be other binding partners of Hco-gal-m and -f participating in the modulation of cell proliferation by rHco-gal-m.

In this study, we found that rHco-gal-m could significantly increase the phagocytosis of monocyte (Figure 5). When *TMEM63A* were knocked down by siRNA, the influence of rHco-gal-m on the induction of phagocytosis in the ns siRNA-treated cells was disrupted (Figure 5). Our findings indicated that the interaction of Hco-gal-m with TMEM63A was involved in the regulation of cell phagocytosis.

Sano et al. found that galectin-3 induced human monocyte migration *in vitro* as a chemo attractant by binding to unknown receptor [47]. In previous transcriptional and proteomic studies, vimentin and coronin-1A, which played a critical role in the attachment and migration of lymphocytes [48,49], was dramatically down-regulated in rHco-gal-m/f-treated PBMC. The further migration assays additionally proved the decreased mobility of rHco-gal-m/f-treated PBMC [30]. The decreased capacity of cell migration due to down-regulation of vimentin and coronin-1A induced by rHco-gal-m/f suggested a mechanism by which parasitic galectins contributed to the worms evading host immunity. In the present study, the percentage of migrated PBMC significantly decreased after the treatment of rHco-gal-m in ns siRNA-treated cells, which was consistent with the results of previous studies. Notably, we also noticed that the percentage of migrated PBMC was significantly increased in TMEM63A knockdown cells, indicating that the rHco-gal-m induced suppression of PBMC migration was significantly blocked by the knockdown of *TMEM63A* gene (Figure 7). Considered together, these results revealed that the rHco-gal-m induced suppression of cell migration was not only related to the down-regulation of vimentin andcoronin-1A, but also mediated by the interaction of Hco-gal-m with TMEM63A.

Nitric oxide is synthesised by many cell types involved in immunity and inflammation [50]. It plays an important role in the majority of parasitic infections, including *H. contortus*, by mediating host protection through either direct parasite killing or by limiting parasite growth [51-53]. Zuniga et al. reported that galectin-1 at its lowest concentration was able to down-regulate critical mediators for *T. cruzi* killing, such as nitric oxide [54]. In our previous analysis, rHco-gal-m/f-treatment decreased the transcription and expression of inducible NOS2A, neutrophil cytosolic factor 1, dualoxidases and carbonic anhydrases 2, which were functional in the production of nitric oxide and reactive oxygen species [30]. So we detected the nitric oxide production in the goat PBMC treated with TMEM63A siRNA and rHco-gal-m here. Incorporation of rHco-gal-m significantly suppressed nitric oxide production in the ns siRNA/g group compared with ns siRNA group (Figure 6). After achieving *TMEM63A* gene knockdown, the nitric oxide production in the 63AsiRNA/g group was significantly increased compared to the cells in the ns siRNA/g group (Figure 6). It showed that influence of rHco-gal-m on the induction of nitric oxide production could be inhibited by TMEM63A knockdown. These results indicated that the interaction of Hco-gal-m with TMEM63A was involved in the regulation of nitric oxide production.

Cytokine secretion plays an important role in immune responses [55,56]. We selected IL-10, IFN-γ and TGF-β1 to represent an anti-inflammatory, Th1 and Treg cytokine, respectively. Our previous research showed that the mRNA transcript levels of IL-10 and TGF-β1 were significantly increased by rHco-gal-m and -f compared to the levels in a control group [30]. In this study, rHco-gal-m could also enhance the expression of IL-10 and TGF-β1 in ns siRNA-treated cells, and these effects were significantly diminished by the knockdown of *TMEM63A* (Figure 8). Previous research found that the mRNA transcription level of IFN-γ in goat PBMCs could be inhibited by rHco-gal-m and -f [30]. In the present study, the IFN-γ profile in ns siRNA-treated cells was also consistent with previous report. Meanwhile, significant increase of IFN-γ mRNA transcripts was detected in the *TMEM63A* knockdown cells stimulated by rHco-gal-m compared to the siRNA-treated cells (Figure 8B). These findings indicated that the influences of rHco-gal-m on IL-10, IFN-γ and TGF-β1 transcription might depend on the binding ability of rHco-gal-m to TMEM63A. Recently, the recombinant galectin of *Toxascarisleonina* (rTl-GAL) was also found to be able to inhibit Th1 and Th2 cytokine production by increasing the production of IL-10 and TGF-β1 [16], suggesting that the influences of galectins from different nematodes on the expression of multiple cytokines in host immunocyte might be similar. Whether the influences of other nematode galectins on cytokine expression also depend on the binding ability of galectin to TMEM63A should be investigated further.

Additionally, we found no significant difference in the levels of IL-10 mRNA transcripts between ns siRNA and TMEM63A knockdown PBMCs in PBS-treated groups (Figure 8A). However, the levels of IFN-γ and TGF-β1 mRNA transcripts in the PBS-treated group in the TMEM63A siRNA-treated cells were significantly regulated compared to the levels in the ns siRNA-treated cells (Figure 8B and C). These results indicated that TMEM63A could not regulatethe production of IL-10 alone, but it might regulate the production of IFN-γ and TGF-β1 directly. However, the mechanisms of TMEM63A directly regulating the transcription of IFN-γ and TGF-β1 should be studied further.

PBMC contain many subtypes of cells. Using flow cytometry, our data indicated that TMEM63A was expressed by most T cells, B cells and monocytes in goat PBMCs (Figure 3). These findings suggested that it might carry out stable and conserved physiological functions in mammalian cells. However, different PBMC subsets have distinct physiological functions. Our previous research indicated that rHco-gal-m modulated goat monocytes and T cell function in different patterns [32]. Characterizing the specific functions of TMEM63A expressed by specific cell subsets requires further research.

Analysis of amino acid sequences of Hco-gal-m and -f proved that they belonged to tandem repeat galectin subfamily which contained mammalian galectin-4, 6, 8, 9 and 12 [4,57]. A growing body of evidence from recent studies indicated that tandem-repeat type galectins from mammalian could bind to appropriate receptors on the cell surface and played various roles in apoptosis, chemo attraction, cell adhesion, cell proliferation, cytokine secretion and immune responses [4]. The galectin-4 secreted by intestinal epithelial cells could bind to CD3 epitope and resulted in the inhibition of T cell activation, cycling and expansion [8]. Galectin-8 interacted with several members of the integrin family and thus regulated cell adhesion and cell survival [9]. Simultaneously, galectin-8 served as a versatile receptor for vesicle-damaging pathogens in the cytosol [10]. Galectin-9 required complex *N*-glycans receptors to kill thymocytes, peripheral T cells, and T cell lines [11]. It was also found that galectin-9 bound to cell surface protein disulfide isomerase on Th2 cells and increased cell migration [58]. Previous research suggested that T cell immunoglobulin domain and mucin domain (Tim)-3 was the receptor of galectin-9 on Th1 cell [59,60], however, a recent study precluded this possibility [61]. Taken together, these findings suggested that different type of galectins, even in the same subfamily, recognized different cell surface receptors and then participated in various biological processes. Our results firstly showed that TMEM63A presented on the cell surface was a binding partner or receptor of Hco-gal-m and -f. Our research filled the gap in the identification of nematode galectin receptor on the host cells.

## Conclusion

In summary, we showed for the first time that TMEM63A was a novel binding partner for Hco-gal-m and -f which presented on the cell surface. The interaction of Hco-gal-m with TMEM63A plays crucial roles in proliferation, phagocytosis, nitric oxide production, migration and cytokines transcription in goat PBMC. These results will not only contribute to understanding the functions of Hco-gal-m and -f, but might also help to elucidate the general mechanisms involved in immune evasion by nematodes and in parasite-host interactions. However, more detailed biological functions of TMEM63A and other binding partners of Hco-gal-m and -f, along with their downstream binding molecules and associated signaling pathways should be further studied.

## Additional files

Additional file 1:
**Supporting Protocol.**
Additional file 2:
**Supporting Tables. Table S1.** Primer sequences for yeast two-hybrid screening. **Table S2.** Primer sequences for PCR amplification. **Table S3.** siRNA sequences for gene knockdown. **Table S4.** Primer sequences for real-time PCR. Additional file 3: Figure S1. N-terminal signal peptide prediction. The amino acid sequences of TMEM63A and Hco-gal-m (NCBI accession numbers: KF850508 and AY253330) were used to predict N-terminal signal peptides by SignalP 4.1 Server. (A): TMEM63A. (B): Hco-gal-m. No protein encoded a predicted N-terminal signal peptide. Additional file 4: Figure S2. Purification of recombinant TMEM63A and Hco-gal-m. Purified recombinant proteins were resolved on 15% acrylamide gels (A and B), and stained with coomassie brilliant blue R250. A: Recombinant TMEM63A-N-terminal protein was approximately 12.37 kDa (including 7 kDa fusion proteins and a 5.37 kDa TMEM63A-N-terminus). B: Recombinant Hco-gal-m protein was approximately 39.50 kDa (including 7 kDa fusion proteins and 32.50 kDa Hco-gal-m). Additional file 5: Figure S3. Confirmation of polyclonal antibody specificity by western blot. Goat PBMCs were lysed with lysate buffer, and loaded in gels in SDS loading buffer. The cell lysates (A and B) or recombinant Hco-gal-m without fusion proteins (C) were resolved on 12% acrylamide gels (A, B and C). Proteins on gels were transferred onto 0.2 μm PVDF transfer membranes and were probed by incubating with anti-TMEM63A-NO IgG (A) or anti-Hco-Gal IgG (B and C) primary antibodies. (A): TMEM63A can be recognized by anti-TMEM63A-NO IgG, and the band was approximately 91.81 kDa. (B): No band was observed in the cell lysates stained by anti-Hco-Gal IgG. (C): Recombinant Hco-gal-m without fusion proteins (the 4.91 kDa fusion proteins were cleaved by thrombin) could be recognized by anti-Hco-Gal IgG and was approximately. Additional file 6: Figure S4. Membrane protein prediction using TMHMM Server v.2.0. The amino acid sequences of TMEM63A (NCBI accession numbers: KF850508) were analyzed to predict transmembrane structures using TMHMM Server v.2.0. The proteins were predicted to contain transmembrane domains. Additional file 7: Figure S5. The knockdown efficiency of TMEM63A at different time points. Goat PBMCs were transfected with TMEM63A siRNA. The level of TMEM63A mRNA transcript was reduced at 48 h (A) after RNAi treatment. An asterisk indicates that the value was significantly different (p < 0.05) from that of the 0 h group. A significant decrease in TMEM63A protein level was observed by western blotting at 48 h after RNAi (B, TMEM63A). Lane 1 to 4 were loaded with cell lysates (10 μg/lane) harvested at 0, 24, 48 and 60 h after RNAi. Beta-actin was used as a protein-loading control (B, Beta-actin). The results presented here are from one independent experiment and are representative of three independent experiments.
