# Supplementary material for: Transmembrane protein 63A is a partner protein of Haemonchus contortus galectin in the regulation of goat peripheral blood mononuclear cells
Source: Parasit Vectors. 2015 Apr 9;8:211. doi: 10.1186/s13071-015-0816-3 (PMC4404006; doi:10.1186/s13071-015-0816-3)
Supplement: Additional file 1: — Supporting Protocol. [file 13071_2015_816_MOESM1_ESM.docx]

**Supporting Protocol**

**1 Construction of a goat PBMC cDNA library for yeast two hybrid screening**

Goat PBMC total RNA was isolated and purified using an E.Z.N.A. total RNA Kit (Omega Bio-tek, Georgia, USA) according to the manufacturer’s instructions. Samples were quantified by spectrophotometry using a Nanotrop2000 (Thermo Fisher Scientific, MA, USA). Genomic DNA contamination in the RNA preparations was removed by treatment with RNase-free DNase I (TaKaRa, Clontech Laboratories, CA, USA). Then, RNA was reverse-transcribed using a cDNA Library Construction Kit (Dualsystems Biotech, Schlieren, Switzerland) according to the manufacturer’s instructions. The cDNA greater than 0.5 kb were selected by size fractionation using CHROMA SPINTM-400 (Clontech Laboratories, CA, USA). In brief, the library was created by ligating cDNA fragments into the pray plasmid pPR3-N (Dualsystems Biotech, Schlieren, Switzerland) using a cDNA library kit (Dualsystems Biotech, Schlieren, Switzerland), according to the manufacturer’s instructions. To confirm the presence of inserts in clones, plasmid DNA was extracted from 20 clones and a restriction digest was performed using SfiI to release the cDNA insert.

**2 Expression of recombinant proteins**

Because no antibody was available, polyclonal antibodies were required. Such antibodies were generated using recombinant protein as antigen. The fragment encoding Hco-gal-m was PCR amplified from the recombinant plasmid pBV220-Hco-gal-m that was constructed in our laboratory (Beck et al., 2003).

TMEM63A was predicted to represent integral membrane proteins; full-length forms of this protein cannot be readily expressed in *E. coli* expression systems. According to our prediction of membrane proteins using TMHMM Server v.2.0 (http://www.cbs.dtu.dk/services/TMHMM/; Figure S4), partial region of TMEM63A was chosen for expression. The partial insert fragment of TMEM63A was PCR amplified using cDNA from goat PBMC, as described previously.

Primer sequences for PCR amplification are listed in the supporting information (Table S2). After confirming the sequence of the PCR product, TMEM63A and Hco-Gal-m fragments were extracted for ligation into the pET-28a(+) vector (Novagen, Merck, Darmstadt, Germany). Following ligation, the constructed plasmids were transformed into *E. coli* strain BL21 (DE3). Recombinant proteins were induced in cells using 1 mM isopropyl-β-d-thiogalactoside (IPTG) treatment. Proteins with His-tags were then purified using a fast protein liquid chromatograph equipped with an AKTA FPLC system (GE Healthcare, NJ, USA). SDS-PAGE gels were used to confirm the size and purity of the recombinant proteins (Figure S2). Thrombin Kits (Novagen, Merck, Darmstadt, Germany) were used to cleave recombinant fusion proteins. After removing recombinant fusion proteins, lipopolysaccharide (LPS) was depleted from the proteins using the Detoxi-Gel Affinity Pak prepacked columns (Pierce, WI, USA), in accordance with the manufacturer’s instructions. Protein concentrations were determined using the Pierce^TM^ BCATM Protein Assay (Thermo Fisher Scientific, MA, USA) and were then diluted to 1 mg/mL using PBS. Endotoxin concentrations were measured using the LAL gel clot assay (Associates of Cape Cod Inc., MA, USA), which showed that endotoxin concentrations for all recombinant proteins were less than 0.25 EU/mL. Ample aliquots were stored at –70 °C until use.

**3 Production of antibody**

SD rats (body weight, ~150 g) were purchased from the Experimental Animal Center of Jiangsu, PR China (Qualified Certificate: SCXK 2008-0004) and were raised in a sterilized room and fed sterilized food and water.

The rats were immunized with 100 μg protein four times at 15-day intervals. Negative serum was collected before the first immunization. The polyclonal antisera were collected 7 days after the final immunization. Rat antisera were subjected to 50% ammonium sulfate precipitation, using a published techniques (Yanming et al., 2007). The precipitated fraction was re-suspended in PBS (pH 7.4) for 24 h at 4°C, including three changes of buffer during this time period. PD MidiTrap G-25 (GE Healthcare, NJ, USA) was used to desalt and adjust this crude preparation to the optimal pH and ionic binding buffer strength (20 mM sodium phosphate, pH 7.0) for IgG purification. Rat IgG was then purified using HiTrap Protein G HP (GE Healthcare, NJ, USA) following the manufacturer’s instructions. Rat IgG concentrations were determined using the Pierce^TM^ BCATM Protein Assay (Thermo Fisher Scientific, MA, USA). Purified rat IgGs, including preimmune IgG and specific IgG, were each diluted to 0.5 mg/mL. Then, IgG aliquots were stored at –70 °C until use. The specificities of IgGs were determined by immunoblotting (Figure S3). The binding specificity of the antibody was further investigated and confirmed by mass-spectometry.

**4 Transfection procedures for siRNA**

For each siRNA transfection, 1×10^6^ cells/well were cultured in 12-well plates. The cells were transfected with 40 pmol TMEM63A-siRNA-1 along with 2 μL Lipofectamine^®^ RNAiMAX Reagent (Life Technologies, USA). In the negative control group, cells were transfected with 40 pmol non-specific siRNA (ns siRNA) and 2 μL Lipofectamine® RNAiMAX Reagent.

To determine the optimal time to achieve maximal interference, cells were harvested at 24, 48 and 60 h after siRNA transfection. Cells were split to two aliquots, one aliquot was lysed using lysis buffer for western blot analysis, and the other aliquot was used for total RNA extraction to confirm the knockdown of target genes using RT-PCR analysis. The mRNA expression levels of beta-actin were used as an internal control and the stability of beta-actin expression was validated. The mouse monoclonal antibodies against beta-actin (1:200) and chicken anti-mouse IgG-HRP (1:3000) were purchased from Santa Cruz Biotechnology (Santa Cruz Biotechnology, Texas, USA). Each experiment was performed in triplicate.

**5 The real-time PCR reactions and conditions**

The real-time PCR reactions were carried out in 96-well optical reaction plates (BioRad Laboratories, CA, USA) with 200 nM each specific primer, 2 μl cDNA (20 ng, as described in the experimental procedures), and SYBR® Green Supermix (BioRad Laboratories, CA, USA) using an ABI 7500 Real-Time PCR system (Applied Biosystems, CA, USA). The PCR conditions were as follows: 95°C for 30 sec, followed by 95°C for 15 sec, 60°C for 30 sec and 72°C for 30 sec for 40 cycles.

**Supplemental References**

Beck, B.M., Rice, C.D., 2003. Serum antibody levels against select bacterial pathogens in Atlantic bottlenose dolphins, Tursiops truncatus, from Beaufort NC USA and Charleston Harbor, Charleston, SC, USA. Mar Environ Res 55, 161-179.

Yanming, S., Ruofeng, Y., Muleke, C.I., Guangwei, Z., Lixin, X., Xiangrui, L., 2007. Vaccination of goats with recombinant galectin antigen induces partial protection against Haemonchus contortus infection. Parasite Immunol 29, 319-326.
