# Supplementary material for: Transmembrane protein 63A is a partner protein of Haemonchus contortus galectin in the regulation of goat peripheral blood mononuclear cells
Source: Parasit Vectors. 2015 Apr 9;8:211. doi: 10.1186/s13071-015-0816-3 (PMC4404006; doi:10.1186/s13071-015-0816-3)
Supplement: Additional file 2: — Supporting Tables. Table S1. Primer sequences for yeast two-hybrid screening. Table S2. Primer sequences for PCR amplification. Table S3. siRNA sequences for gene knockdown. Table S4. Primer sequences for real-time PCR. [file 13071_2015_816_MOESM2_ESM.docx]

**Supporting Tables**

**Table S1. Primer sequences for yeast two-hybrid screening**

| Primer Name | Sequence (5’-3’) |
| --- | --- |
| Hco-Gal-F | CCTGCATggccattacggccGCCACCATGGTGTCACAGTTCCTACACTGG (*Sfi* I) |
| Hco-Gal-R | TATCGACggccgaggcggccCTGGATCTGGATGCCAGTCAG (*Sfi* I) |
| pPR3N-F | GTCGAAAATTCAAGACAAGG |
| pPR3N-R | AGCGTGACATAACTAATTAC |

**Table S2. Primer sequences for PCR amplification**

| Gene Name | Primer Sequence (5’-3’) |
| --- | --- |
| Hco-Gal-m | GCGgaattcATGGTGTCACAGTTCCTAC (*Eco*R I) |
|  | TATgtcgacCTACTGGATCTGGATGCC (*Sal* I) |
| TMEM63A | TTCgaattcCACCACACCCAGTCCATCAAG (*Eco*R I) |
|  | GATaagcttTCATTGGAACCACCAGCGGAAGC (*Hind* III) |

**Table S3. siRNA sequences for gene knockdown**

| Gene Name | Primer Sequence (5’-3’) |
| --- | --- |
| TMEM63A-siRNA-1 | GCAUCAUCCUGCCUGUCAATT |
|  | UUGACAGGCAGGAUGAUGCTT |
| TMEM63A-siRNA-2 | CCAUUAUCCUGUCCACCAUTT |
|  | AUGGUGGACAGGAUAAUGGTT |
| TMEM63A-siRNA-3 | GCGUCUUCACCGUCAUCAUTT |
|  | AUGAUGACGGUGAAGACGCTT |
| Non-specific siRNA | UUCUCCGAACGUGUCACGUTT |
|  | ACGUGACACGUUCGGAGAATT |

**Table S4. Primer sequences for real-time PCR**

| Gene Name | | Primer Sequence (5’-3’) | Amplification efficiency (%)* | Correlation coefficients (r^2^) |
| --- | --- | --- | --- | --- |
| beta-actin | | CACCACACCTTCTACAAC | 95.41 | 0.9991 |
|  | TCTGGGTCATCTTCTCAC | |  |  |
| TMEM63A | | CTGGCTCTACTTCTTCTCCTTC | 97.68 | 0.9994 |
|  | | CCGATTCTTCTGTCTTGTAGTTG |  |  |
| IL-10 | CCTTGTCGGAAATGATCCAG | | 98.68 | 0.9993 |
|  | AGGGCAGAAAACGATGACAG | |  |  |
| IFN-γ | GAACGGCAGCTCTGAGAAAC | | 98.02 | 0.9982 |
|  | GGTTAGATTTTGGCGACAGG | |  |  |
| TGF-β1 | CATGAACCGGCCCTTCCT | | 98.98 | 0.9996 |
|  | GAAGTCAATGTAGAGCTGACGAACA | |  |  |

^*^ Amplification efficiency (%) = (10^-1/slope^ -1) ×100
