# Supplementary figures and images for: Transmembrane protein 63A is a partner protein of Haemonchus contortus galectin in the regulation of goat peripheral blood mononuclear cells
Source: Parasit Vectors. 2015 Apr 9;8:211. doi: 10.1186/s13071-015-0816-3 (PMC4404006; doi:10.1186/s13071-015-0816-3)

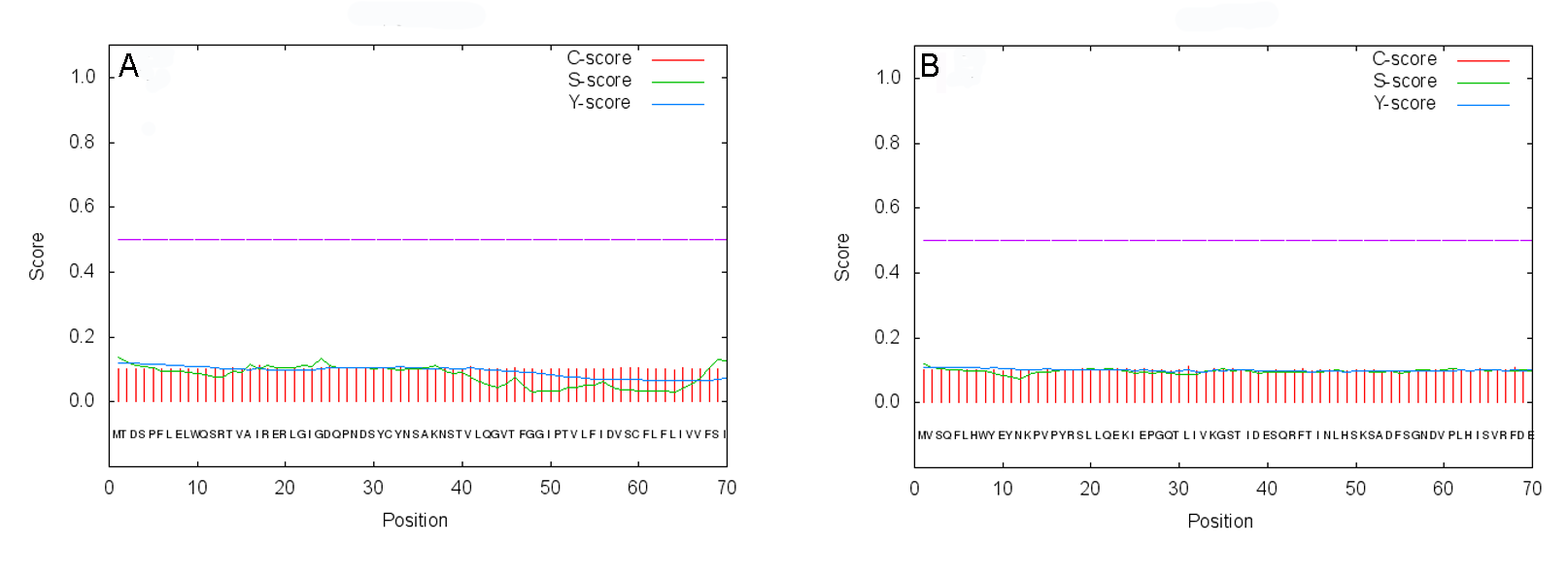

Supplement: Additional file 3: Figure S1. — N-terminal signal peptide prediction. The amino acid sequences of TMEM63A and Hco-gal-m (NCBI accession numbers: KF850508 and AY253330) were used to predict N-terminal signal peptides by SignalP 4.1 Server. (A): TMEM63A. (B): Hco-gal-m. No protein encoded a predicted N-terminal signal peptide. [file 13071_2015_816_MOESM3_ESM.tif]

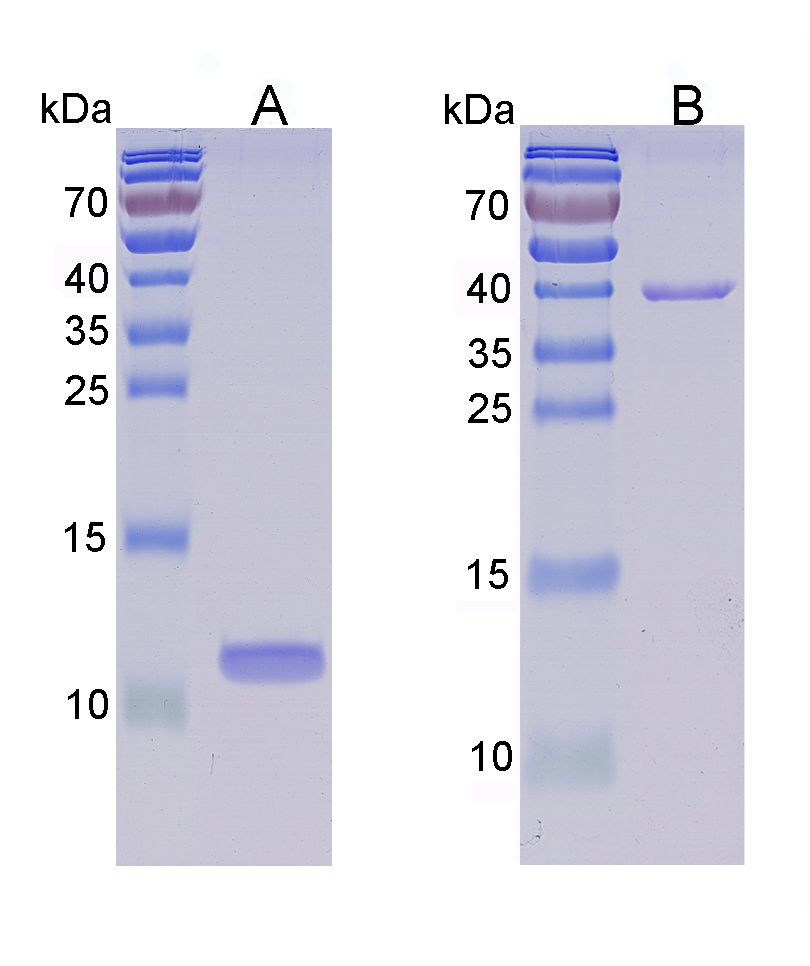

Supplement: Additional file 4: Figure S2. — Purification of recombinant TMEM63A and Hco-gal-m. Purified recombinant proteins were resolved on 15% acrylamide gels (A and B), and stained with coomassie brilliant blue R250. A: Recombinant TMEM63A-N-terminal protein was approximately 12.37 kDa (including 7 kDa fusion proteins and a 5.37 kDa TMEM63A-N-terminus). B: Recombinant Hco-gal-m protein was approximately 39.50 kDa (including 7 kDa fusion proteins and 32.50 kDa Hco-gal-m). [file 13071_2015_816_MOESM4_ESM.tif]

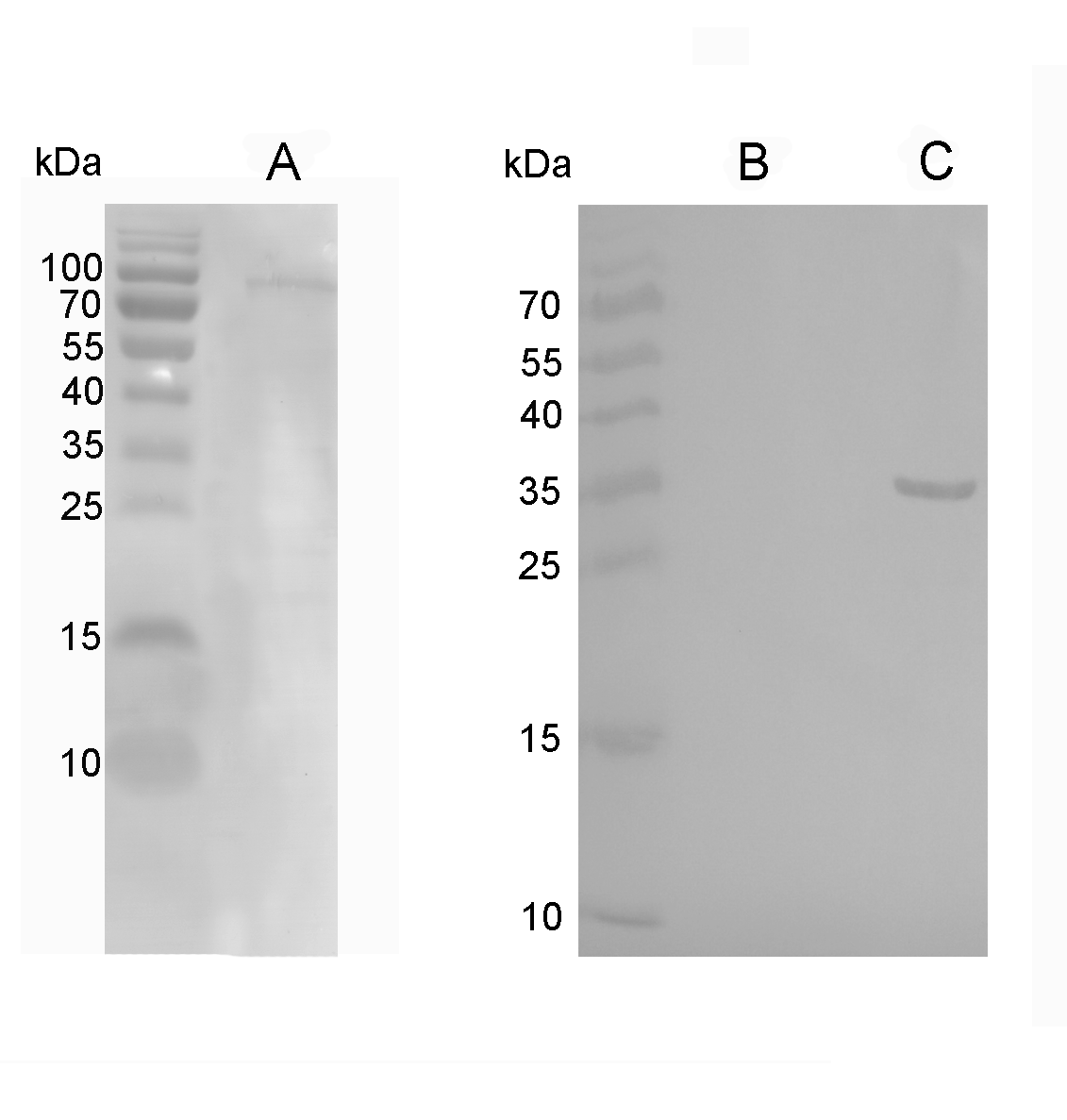

Supplement: Additional file 5: Figure S3. — Confirmation of polyclonal antibody specificity by western blot. Goat PBMCs were lysed with lysate buffer, and loaded in gels in SDS loading buffer. The cell lysates (A and B) or recombinant Hco-gal-m without fusion proteins (C) were resolved on 12% acrylamide gels (A, B and C). Proteins on gels were transferred onto 0.2 μm PVDF transfer membranes and were probed by incubating with anti-TMEM63A-NO IgG (A) or anti-Hco-Gal IgG (B and C) primary antibodies. (A): TMEM63A can be recognized by anti-TMEM63A-NO IgG, and the band was approximately 91.81 kDa. (B): No band was observed in the cell lysates stained by anti-Hco-Gal IgG. (C): Recombinant Hco-gal-m without fusion proteins (the 4.91 kDa fusion proteins were cleaved by thrombin) could be recognized by anti-Hco-Gal IgG and was approximately. [file 13071_2015_816_MOESM5_ESM.tif]

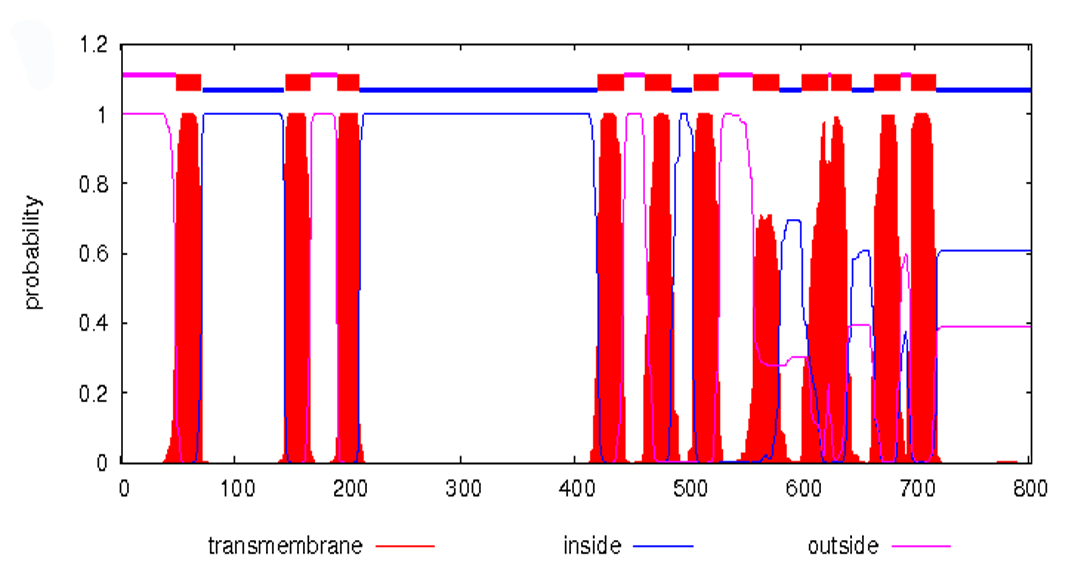

Supplement: Additional file 6: Figure S4. — Membrane protein prediction using TMHMM Server v.2.0. The amino acid sequences of TMEM63A (NCBI accession numbers: KF850508) were analyzed to predict transmembrane structures using TMHMM Server v.2.0. The proteins were predicted to contain transmembrane domains. [file 13071_2015_816_MOESM6_ESM.tif]

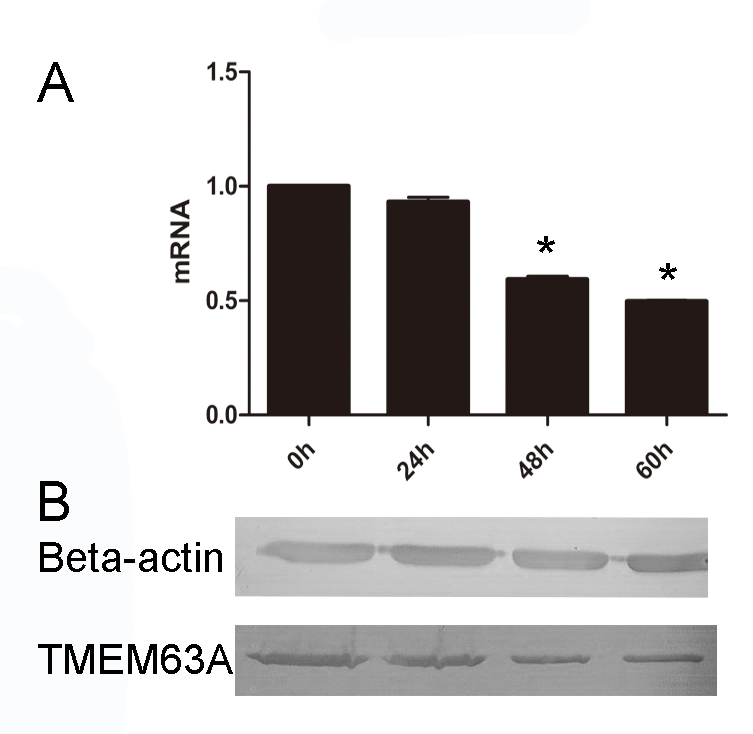

Supplement: Additional file 7: Figure S5. — The knockdown efficiency of TMEM63A at different time points. Goat PBMCs were transfected with TMEM63A siRNA. The level of TMEM63A mRNA transcript was reduced at 48 h (A) after RNAi treatment. An asterisk indicates that the value was significantly different (p < 0.05) from that of the 0 h group. A significant decrease in TMEM63A protein level was observed by western blotting at 48 h after RNAi (B, TMEM63A). Lane 1 to 4 were loaded with cell lysates (10 μg/lane) harvested at 0, 24, 48 and 60 h after RNAi. Beta-actin was used as a protein-loading control (B, Beta-actin). The results presented here are from one independent experiment and are representative of three independent experiments. [file 13071_2015_816_MOESM7_ESM.tif]
